# Supplementary material for: Conformational dynamics of exopolysaccharides underlie biofilm matrix mechanics in Vibrio cholerae
Source: bioRxiv. 2026 Jul 23:2026.07.22.739955. Preprint. [Version 1] doi: 10.64898/2026.07.22.739955 (PMC13420445; doi:10.64898/2026.07.22.739955)
Supplement: Supplement 2 [file NIHPP2026.07.22.739955v1-supplement-2.pdf]

bioRxiv preprint doi: https://doi.org/10.64898/2026.07.22.739955; this version posted July 23, 2026. The copyright holder for this preprint (which was not certified by peer review) is the author/funder, who has granted bioRxiv a license to display the preprint in perpetuity. It is made available under a CC-BY 4.0 International license.

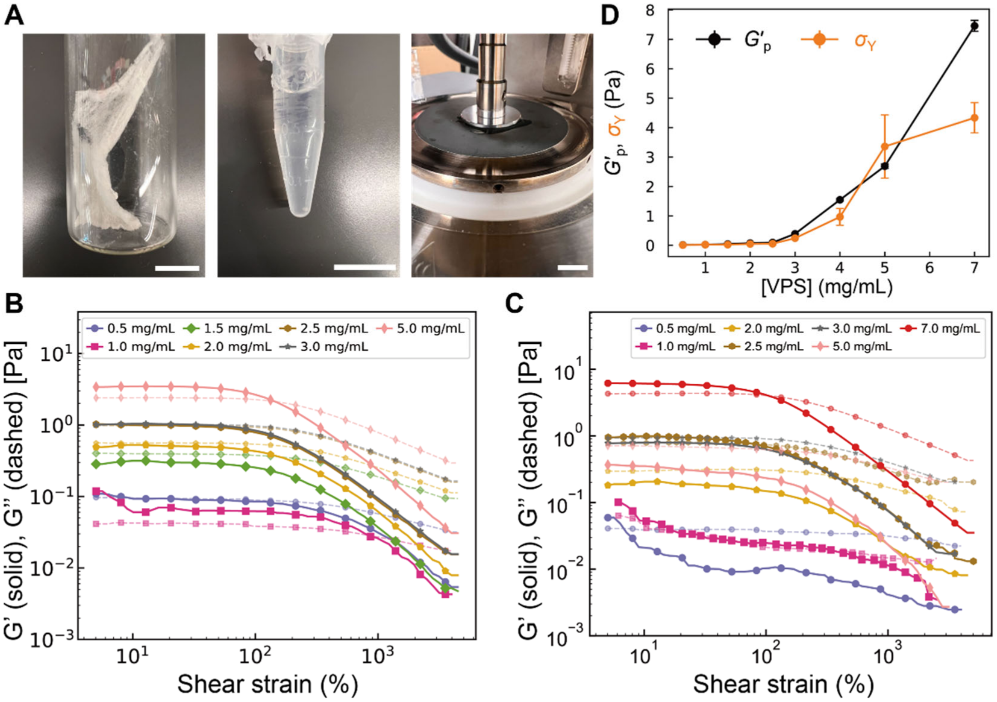

Fig. S1. Rheological measurements of VPS solutions. (**A**) Representative images of purified VPS in solid state form (*top*), in solution (*middle*), and following transfer to the lower plate of a rheometer (*bottom*). Scale bars: 1 cm. (**B** and **C**) G' and G'' versus shear strain for VPS solutions in DI water (**B**) and PBS (**C**) at different concentrations. (**D**) Extracted plateau modulus G'_p and yield stress σ_Y as a function of VPS concentration in PBS.

bioRxiv preprint doi: https://doi.org/10.64898/2026.07.22.739955; this version posted July 23, 2026. The copyright holder for this preprint (which was not certified by peer review) is the author/funder, who has granted bioRxiv a license to display the preprint in perpetuity. It is made available under a CC-BY 4.0 International license.

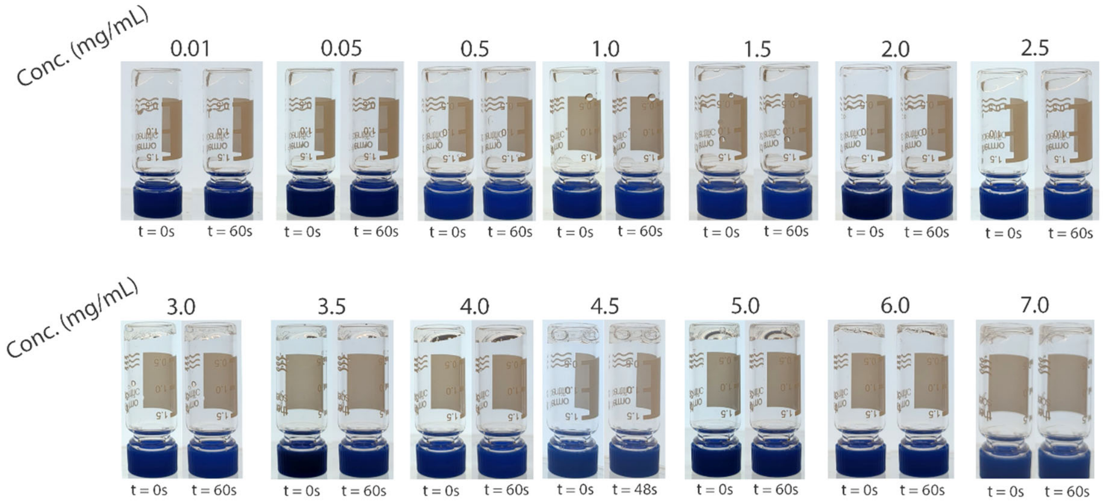

Fig. S2. Pictures of VPS solutions in water at different concentrations, before and after flipping the vials containing them for 60 s. At or below 2 mg/mL, the solution quickly drips to the bottom. Above 2 mg/mL, the solution largely remains at the top.

bioRxiv preprint doi: https://doi.org/10.64898/2026.07.22.739955; this version posted July 23, 2026. The copyright holder for this preprint (which was not certified by peer review) is the author/funder, who has granted bioRxiv a license to display the preprint in perpetuity. It is made available under a CC-BY 4.0 International license.

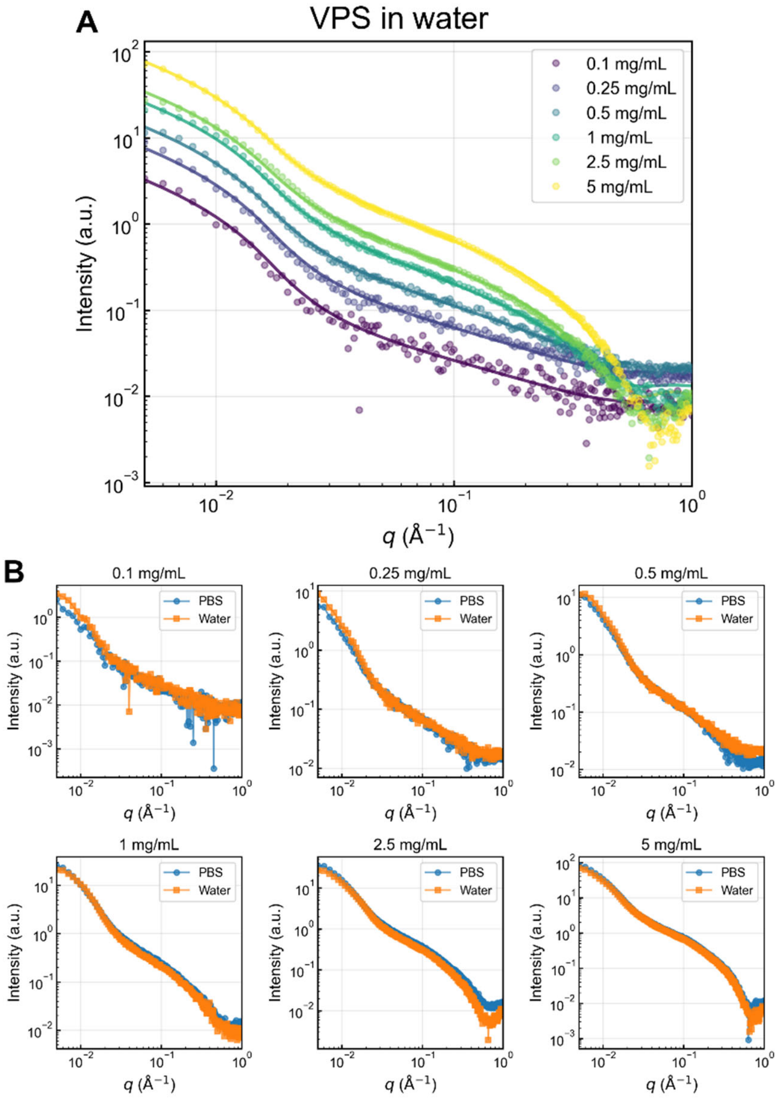

Fig. S3. Additional SAXS data in DI water. (**A**) SAXS intensity versus q for different concentrations of VPS solutions in water. The same fitting procedure was applied to this dataset as in Fig. 3D. (**B**) Comparison between SAXS curves for VPS in water and in PBS at different concentrations.

bioRxiv preprint doi: https://doi.org/10.64898/2026.07.22.739955; this version posted July 23, 2026. The copyright holder for this preprint (which was not certified by peer review) is the author/funder, who has granted bioRxiv a license to display the preprint in perpetuity. It is made available under a CC-BY 4.0 International license.

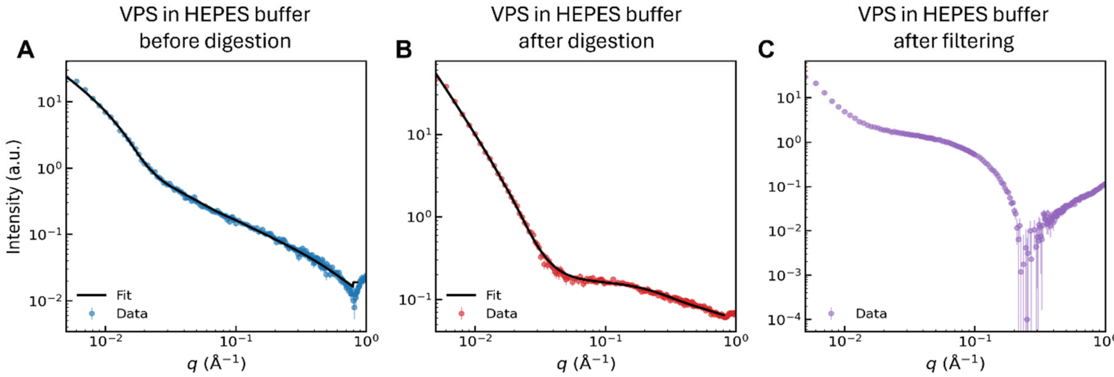

1308
1309 **Fig. S4. The observed features in SAXS curves arise from VPS polymers.** (**A**) SAXS
1310 intensity curve for VPS solution in HEPES buffer, showing similar features to those in Fig. 3D.
1311 (**B**) SAXS curve for VPS solution after enzymatic digestion with RbmB in HEPES buffer into
1312 smaller units. The same fitting procedure was applied to the data in panels **A** and **B** as in Fig.
1313 3D. (**C**) SAXS curve for VPS solution in HEPES buffer after filtering through a solid-phase
1314 extraction column to remove large polymers. No fitting can be appropriately obtained for this
1315 case. All initial concentrations of VPS solutions were ~ 0.5 mg/mL.
1316

bioRxiv preprint doi: https://doi.org/10.64898/2026.07.22.739955; this version posted July 23, 2026. The copyright holder for this preprint (which was not certified by peer review) is the author/funder, who has granted bioRxiv a license to display the preprint in perpetuity. It is made available under a CC-BY 4.0 International license.

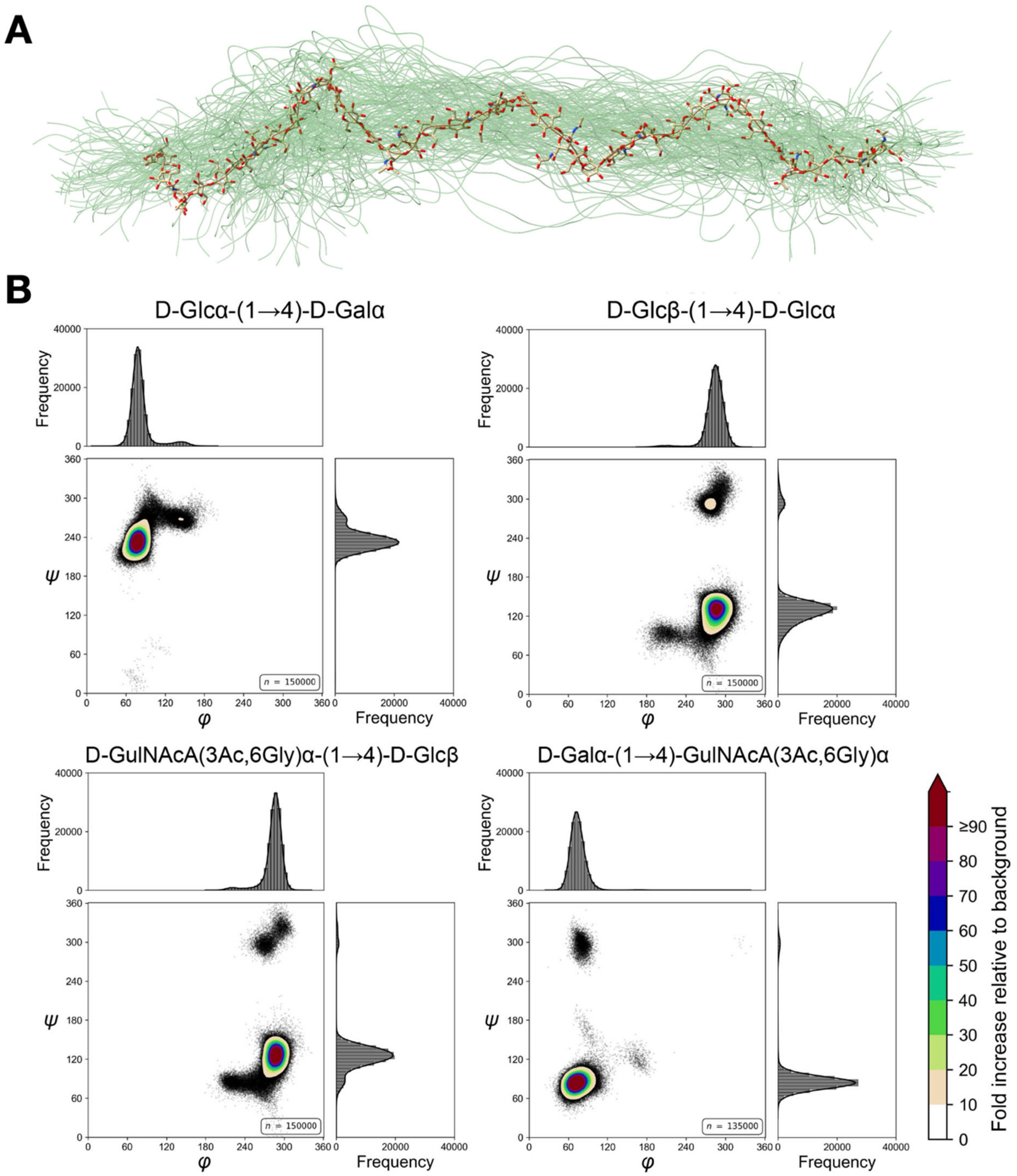

Fig. S5. Dihedral angles for the four glycosidic linkage types. (**A**) Overlaid snapshots from the 10-tetrasaccharide VPS segment in the all-atom MD simulations, pooled over all three replicate trajectories. Each configuration is depicted as a spline interpolating the heavy-atom centroids of the monosaccharide rings along the segment. A representative configuration, namely the configuration with the least root-mean-square deviation to the average positions of the heavy atoms along the segment, is also shown. (**B**) Contour maps of the ϕ (O5–C1–O4'–C4') and ψ (C1–O4'–C4'–C3') dihedral angles along each of the four glycosidic linkage types in the VPS segment. For each linkage type, a histogram of dihedral angles was first computed on a 2D grid of 72 × 72 bins (5° resolution); the background frequency was then defined as the total number of points divided by the total number of bins. Each level set in the contour map represents the bins in the histogram that exceed the indicated fold-increase relative to this background frequency. See also Table S2.

bioRxiv preprint doi: https://doi.org/10.64898/2026.07.22.739955; this version posted July 23, 2026. The copyright holder for this preprint (which was not certified by peer review) is the author/funder, who has granted bioRxiv a license to display the preprint in perpetuity. It is made available under a CC-BY 4.0 International license.

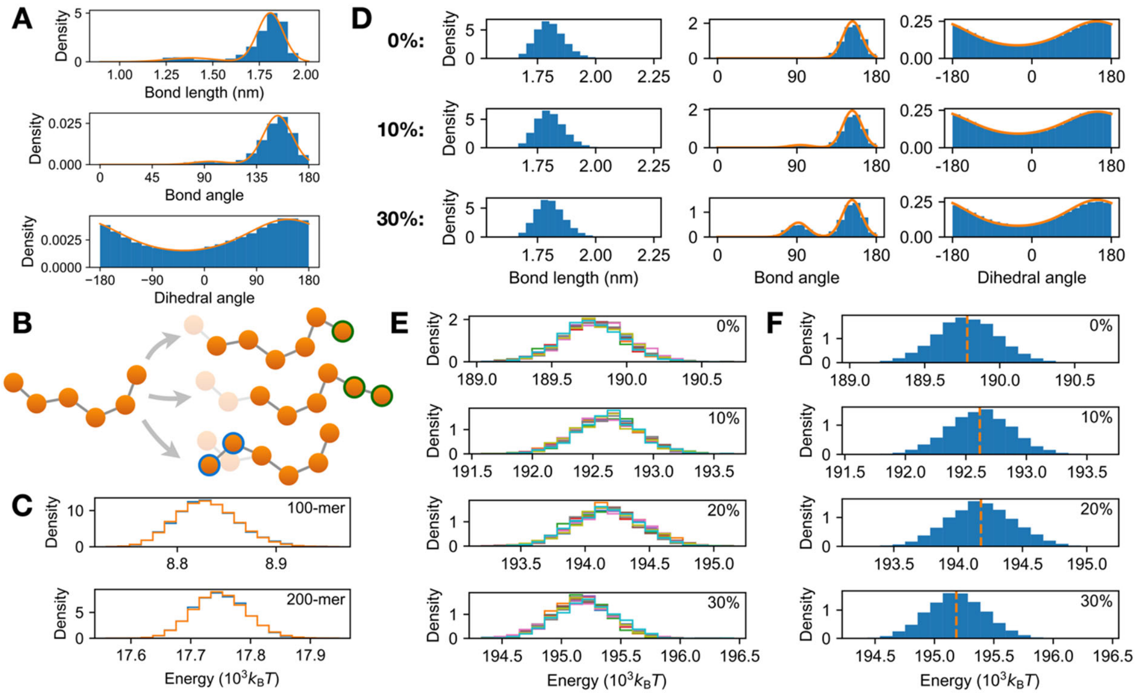

Fig. S6. Coarse-grained VPS model and MC conformational sampling. (**A**) Empirical bond length, bond angle, and dihedral angle distributions along the coarse-grained VPS chain, obtained from each trajectory of the all-atom MD simulations. The bond length distribution was fitted to a two-component Gaussian mixture model; the bond angle distribution was fitted to a symmetrically folded two-component von Mises mixture model; and the dihedral angle distribution was fitted to a von Mises distribution (orange curves; see also Table S3). See Methods for details. (**B**) Schematic of the MC conformational sampling procedure. At each iteration, a set of new candidate polymer configurations was proposed using one of three move types: reptation by a single bead (*top*), reptation by multiple beads (*middle*), or terminal segment move (*bottom*). One candidate configuration was then selected and probabilistically accepted, as prescribed in configurational-bias MC[64]. Transparent beads indicate those in the original configuration that have been deleted or moved; beads with green outlines are new beads that have been introduced through reptation; and beads with blue outlines are pre-existing beads that have been moved. See Methods and Supplementary Information for details. (**C**) Distributions of configurational energy for a 100- or 200-bead random coil (*top* and *bottom*, respectively), obtained either from 10 independent MC runs (blue) or 20 independent MD trajectories (orange). The random coil was assumed to follow the same non-bonded interaction and bond length potentials as in the coarse-grained model for VPS (see Methods and Supplementary Information). All MD trajectories were run with a Langevin thermostat at 300 K for 500 ns each. (**D**) Bond length, bond angle, and dihedral angle distributions from 10 independent MC runs for the indicated kink fractions. Fitting each bond angle distribution to a folded two-component von Mises mixture model yields the desired fractions of linear (~160°) and kinked (~90°) bond angles; fitting each dihedral angle distribution to a von Mises distribution yields the desired mean dihedral angle (~150°) and concentration (~0.5). (**E** and **F**) Distributions of configurational energy from 10 independent MC runs for each kink fraction, shown as separate distributions for each run in panel **E** and as one distribution pooled over all 10 runs in panel **F**. The mean of each distribution in panel **F** is shown in orange. The overall similarity of each group of 10 distributions in panel **E** to each other, and to the corresponding pooled distribution in panel **F**, suggest that the procedure is effectively sampling from a common equilibrium distribution.

bioRxiv preprint doi: https://doi.org/10.64898/2026.07.22.739955; this version posted July 23, 2026. The copyright holder for this preprint (which was not certified by peer review) is the author/funder, who has granted bioRxiv a license to display the preprint in perpetuity. It is made available under a CC-BY 4.0 International license.

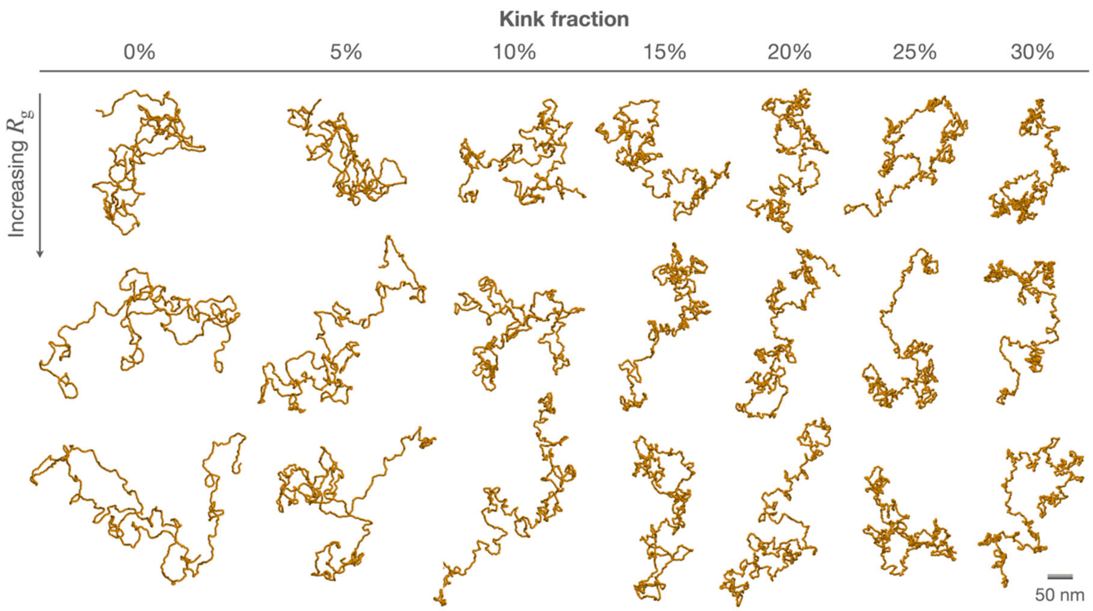

Fig. S7. Example coarse-grained polymer configurations. For each kink fraction, the three configurations with radii of gyration closest to the first, second, and third quartiles (top, middle, and bottom rows, respectively) over the corresponding ensemble are shown. Each set of quartiles was quantified over a pooled ensemble obtained from 10 independent MC runs.

bioRxiv preprint doi: https://doi.org/10.64898/2026.07.22.739955; this version posted July 23, 2026. The copyright holder for this preprint (which was not certified by peer review) is the author/funder, who has granted bioRxiv a license to display the preprint in perpetuity. It is made available under a CC-BY 4.0 International license.

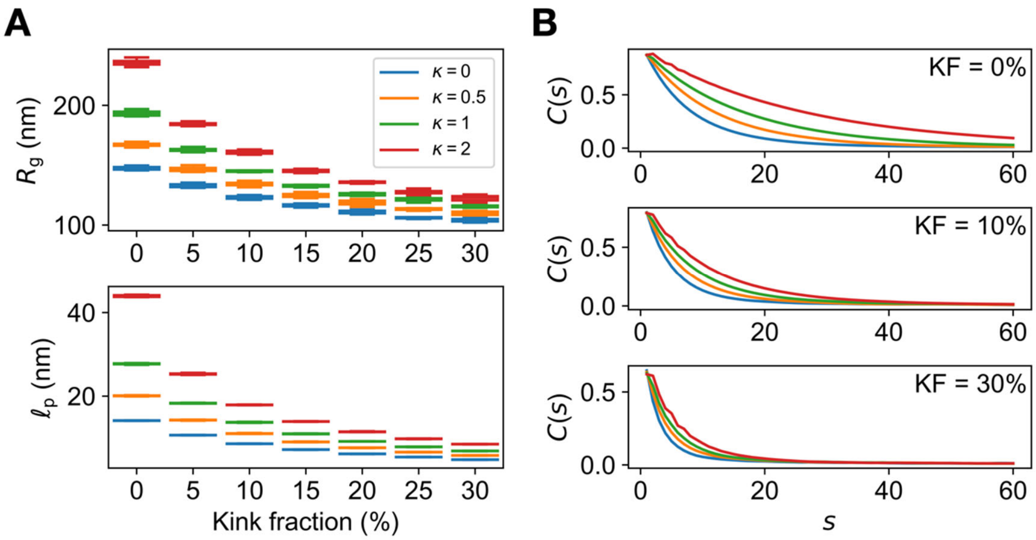

Fig. S8. Effects of dihedral angle stiffness on the radius of gyration and persistence length. (**A**) Root-mean-square radius of gyration (R_g) and persistence length (l_p) as a function of the kink fraction, for different choices of dihedral angle stiffness, $\kappa = K_{\mathrm{dihedral}}/(k_B T)$. $\kappa = 0.5$ corresponds to the coarse-grained model for VPS (Fig. 4F; see also Table S4). Each distribution was obtained from 10 measurements, each taken from an independent MC run for a coarse-grained VPS polymer consisting of 2,100 units. Whiskers correspond to 1.5 times the interquartile range below and above the first and third quartiles, respectively. (**B**) Tangent vector autocorrelation function, $C(s)$, along coarse-grained VPS polymers with the indicated kink fractions and the same values of κ as in panel **A**. The autocorrelation for a fixed value of s was calculated as the average dot product over all pairs of unit tangent vectors s bonds apart along the polymer, averaged over all configurations in the ensemble (see Supplementary Information). Each curve corresponds to an ensemble of configurations obtained from one MC run. We note that, while the dihedral potential imposes some helicity on the VPS polymer (see Methods), a stiffness of $K_{\mathrm{dihedral}} \leq k_B T$ is sufficiently weak that it merely increases the persistence length of the polymer, without imparting any noticeable periodicity to the autocorrelation function; some weak periodicity is apparent at $K_{\mathrm{dihedral}} = 2k_B T$, but the autocorrelation function still appears to be well-described by an exponential decay.

bioRxiv preprint doi: https://doi.org/10.64898/2026.07.22.739955; this version posted July 23, 2026. The copyright holder for this preprint (which was not certified by peer review) is the author/funder, who has granted bioRxiv a license to display the preprint in perpetuity. It is made available under a CC-BY 4.0 International license.

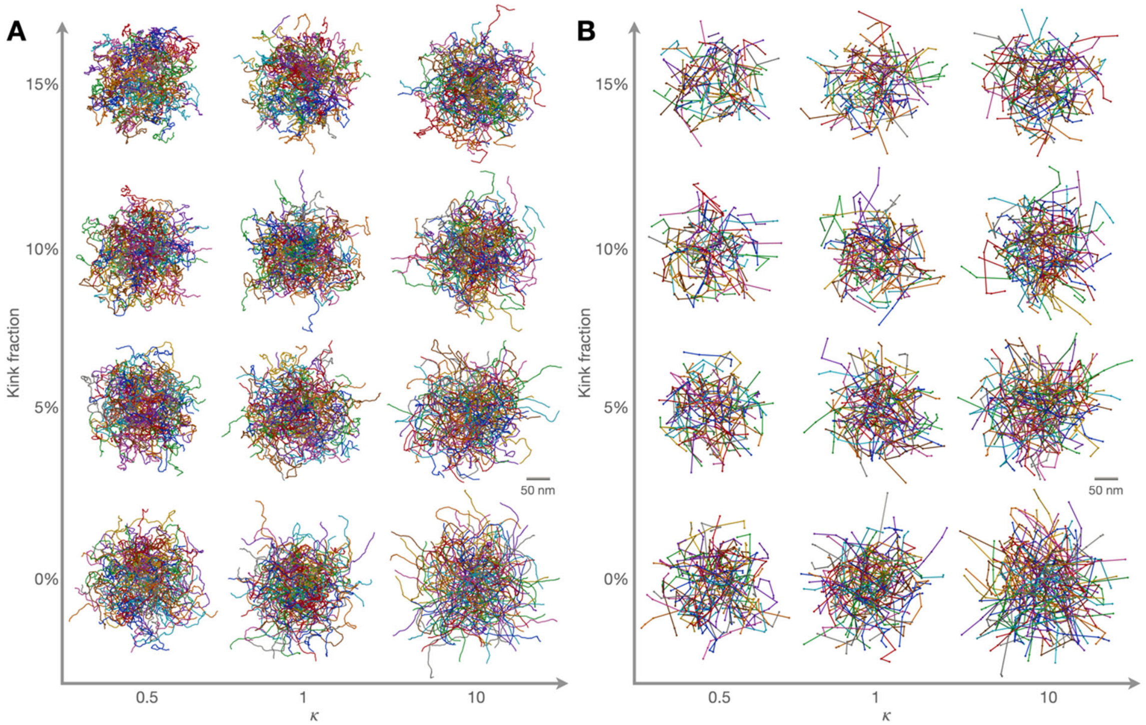

Fig. S9. Primitive path analysis of coarse-grained polymer solution configurations. Representative configurations of solutions of VPS-like chains with the indicated values of kink fraction and dihedral stiffness, $\kappa = K_{\mathrm{dihedral}}/(k_{\mathrm{B}}T)$ (**A**), and the corresponding collections of primitive paths (**B**), each obtained using the Z1+ package. Each chain in panel **A** and each primitive path in panel **B** is plotted such that its center-of-mass lies in the fundamental unit cell in the corresponding periodic domain; as such, a chain and its corresponding primitive path may occasionally be visualized in different positions.

bioRxiv preprint doi: https://doi.org/10.64898/2026.07.22.739955; this version posted July 23, 2026. The copyright holder for this preprint (which was not certified by peer review) is the author/funder, who has granted bioRxiv a license to display the preprint in perpetuity. It is made available under a CC-BY 4.0 International license.

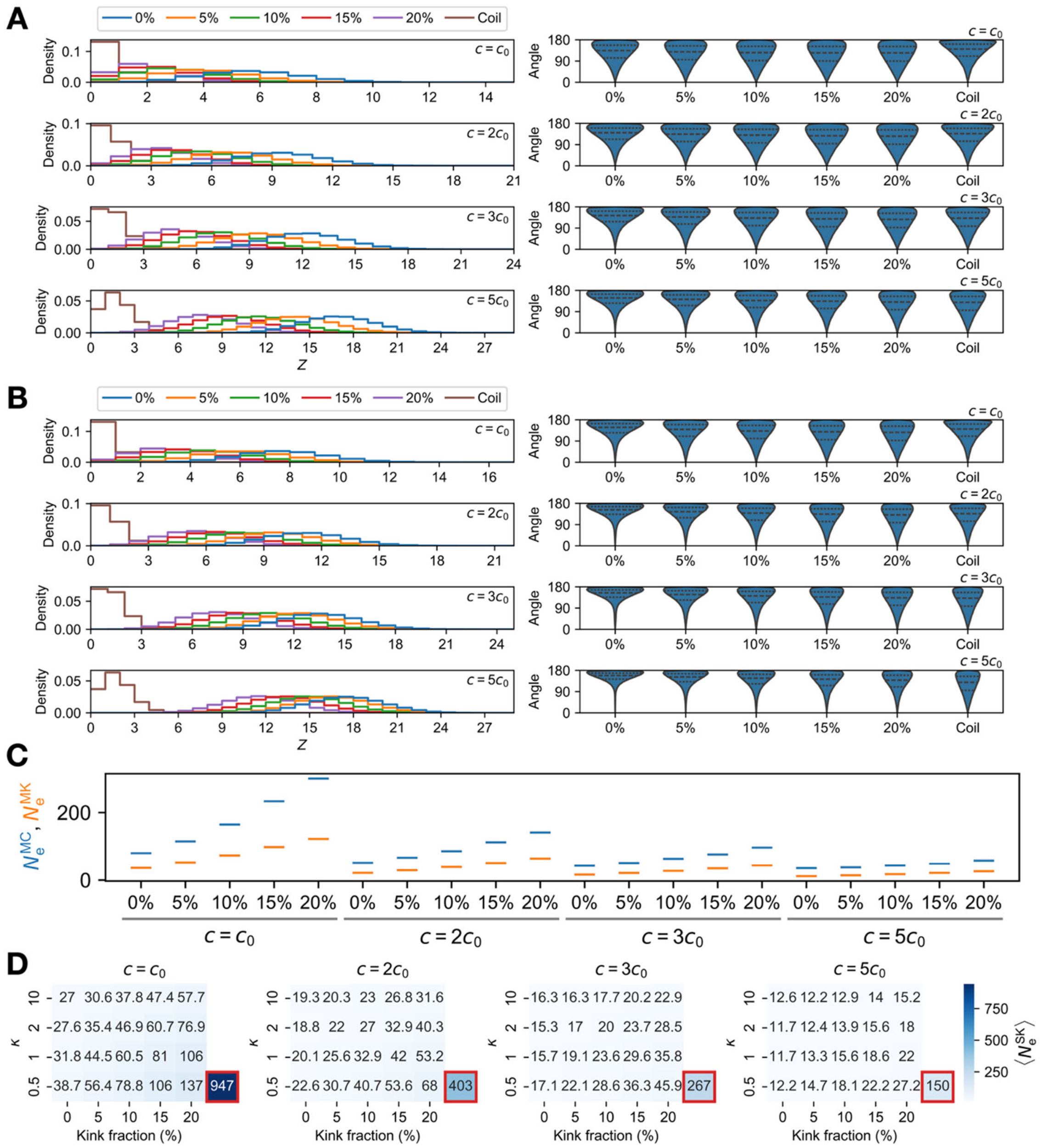

Fig. S10. Entanglement-related quantities for different kink fractions and concentrations. (**A** and **B**) Distributions of the number, Z, of internal nodes along each primitive path (*left*) and the angles formed at these internal nodes (*right*) for each choice of kink fraction and concentration (c), for dihedral stiffnesses of $K_{\text{dihedral}} = 0.5k_{\text{B}}T$ (**A**) and $K_{\text{dihedral}} = 2k_{\text{B}}T$ (**B**). Analogous distributions for solutions of random coils following the same non-bonded interaction and bond length potentials are shown for comparison. (**C**) The "M-coil" and "M-kink" estimators, N_{e}^{MC} and N_{e}^{MK}, for the entanglement length, N_{e}, for $c = c_0, 2c_0, 3c_0, 5c_0$ and $K_{\text{dihedral}} = 0.5k_{\text{B}}T$. We note that N_{e}^{MC} is around twice N_{e}^{MK} for all kink fractions and concentrations, which is consistent with previous work[110]; N_{e}^{MC} better corresponds to the "rheological" entanglement length that determines the plateau modulus. (**D**) The "modified S-kink" estimator, $\langle N_{\text{e}}^{\text{SK}} \rangle$, for $c = c_0, 2c_0, 3c_0, 5c_0$ and $\kappa = K_{\text{dihedral}}/(k_{\text{B}}T) = 0.5, 1, 2, 10$. This estimator is simpler to evaluate than N_{e}^{MC} and N_{e}^{MK}—which require configurations of polymer solutions with different choices of polymer length, N—but is less statistically robust.

bioRxiv preprint doi: https://doi.org/10.64898/2026.07.22.739955; this version posted July 23, 2026. The copyright holder for this preprint (which was not certified by peer review) is the author/funder, who has granted bioRxiv a license to display the preprint in perpetuity. It is made available under a CC-BY 4.0 International license.

**Strain**	**Genotype**	**Source**
JY286	*vpvC*^{W240R}Δ*rbmA*Δ*bap1*Δ*rbmC*Δ*pomA*	[48]
JY462	*vpvC*^{W240R}Δ*rbmA*Δ*bap1*Δ*rbmC*Δ*VC1807*::P_{tac}-*mNeonGreen*, Spec^{R}	[43]
JY477	*vpvC*^{W240R}Δ*rbmA*Δ*bap1*Δ*rbmC*Δ*vpsL*Δ*VC1807*::P_{tac}-*mNeonGreen*, Spec^{R}	[43]

Table S1. List of *V. cholerae* strains used in this paper.

bioRxiv preprint doi: https://doi.org/10.64898/2026.07.22.739955; this version posted July 23, 2026. The copyright holder for this preprint (which was not certified by peer review) is the author/funder, who has granted bioRxiv a license to display the preprint in perpetuity. It is made available under a CC-BY 4.0 International license.

**Glycosidic linkage**	**Stable state**			**Metastable state (%)**	
	ϕ (°)	ψ (°)	**%**	**Anti-ϕ**	**Anti-ψ**
D-Glcα-(1 → 4)-D-Galα	80 ± 8	–125 ± 12	82.8	-	-
D-Glcβ-(1 → 4)-D-Glcα	–71 ± 9	130 ± 13	77.5	-	2.3 (9.5)
L-GulNAcA(3Ac,6Gly)α-(1 → 4)-D-Glcβ	–71 ± 8	128 ± 12	81.9	-	- (4.3)
D-Galα-(1 → 4)-L-GulNAcA(3Ac,6Gly)α	76 ± 10	86 ± 9	88.6	-	- (3.5)

Table S2. Conformational statistics of the four glycosidic linkage types. States and their empirical probabilities were identified according to the contour maps in Fig. S5B; in particular, the empirical probabilities correspond to the fraction of points in (ϕ, ψ)-space that lie within the level sets corresponding to a greater than 10-fold increase over the background frequency. We also quantified the percentage of points near the level set corresponding to the D-Glcβ-(1 → 4)-D-Glcα anti-ψ conformation (~ 7.2%, yielding a total percentage of ~ 9.5%), as well as the percentages of points that may represent analogous anti-ψ conformations for the L-GulNAcA(3Ac,6Gly)α- (1 → 4) -D-Glcβ and D-Galα- (1 → 4) -L-GulNAcA(3Ac,6Gly)α linkages, but did not meet the 10-fold threshold.

bioRxiv preprint doi: https://doi.org/10.64898/2026.07.22.739955; this version posted July 23, 2026. The copyright holder for this preprint (which was not certified by peer review) is the author/funder, who has granted bioRxiv a license to display the preprint in perpetuity. It is made available under a CC-BY 4.0 International license.

**Bond lengths (major component only, weight = 87.1%)**		
Mean		1.81 nm
Standard deviation		0.0690 nm
**Bond angles**		
Peak 1	Mean	153°
	Concentration	21.5
	Weight	92.7%
Peak 2	Mean	95.2°
	Concentration	15.0
	Weight	7.3%
**Dihedral angles**		
Mean		144°
Concentration		0.523

Table S3. Bond length, bond angle, and dihedral angle statistics for the coarse-grained VPS chain. Only the major component of the bond length mixture model is specified.

bioRxiv preprint doi: https://doi.org/10.64898/2026.07.22.739955; this version posted July 23, 2026. The copyright holder for this preprint (which was not certified by peer review) is the author/funder, who has granted bioRxiv a license to display the preprint in perpetuity. It is made available under a CC-BY 4.0 International license.

Parameter	Meaning	Value
T	Temperature	300 K
$\langle l_0 \rangle$	Mean bond length	1.8 nm
σ	Non-bonded interaction length scale	$\langle l_0 \rangle/0.965 \approx 1.865$ nm
k_{FENE}	Bond stiffness	$(9\ \mathrm{nm}^{-2}) \cdot k_{\mathrm{B}}T$
R_0	Maximum bond length	$1.5\sigma \approx 2.798$ nm
w_1	Two times the standard deviation of the larger bond angles in the bimodal angle potential	$\sqrt{1/5} \approx 0.4472$
w_2	Two times the standard deviation of the smaller bond angles in the bimodal angle potential	$\sqrt{1/5} \approx 0.4472$
K_{dihedral}	Dihedral angle stiffness	$0.5k_{\mathrm{B}}T$
δ	Dihedral angle offset	$-30°$

Table S4. Parameters for MC conformational sampling of coarse-grained VPS chains. See Supplementary Information for a full discussion of the parameters and their definitions. To set the Lennard-Jones and FENE potential parameters (σ, k_{FENE}, and R_0), we first fixed $\sigma = \langle l_0 \rangle/0.965 \approx 1.865$ nm and $R_0 = 1.5\sigma \approx 2.798$ nm, as in Svaneborg and Everaers' modified Kremer–Grest model[62,63], and set k_{FENE} so as to obtain a Boltzmann distribution with mean bond length $\langle l_0 \rangle = 1.8$ nm. This yielded an optimal value of $k_{\mathrm{FENE}}/(k_{\mathrm{B}}T) \approx 9.432\ \mathrm{nm}^{-2}$, and so we set $k_{\mathrm{FENE}}/(k_{\mathrm{B}}T) = 9\ \mathrm{nm}^{-2}$. To set w_1 and w_2, we recalled that a von Mises distribution with a large concentration parameter, $\kappa \gg 1$, is approximately Gaussian with variance $\sigma^2 = 1/\kappa$. As such, using the concentration parameter of ~ 20 for the major component in the bond angle mixture in Table S3, we set $w_1^2/4 = 1/20$, or $w_1 = \sqrt{1/5} \approx 0.4472$; for simplicity, we set $w_2 = w_1$. Finally, we set $K_{\mathrm{dihedral}} = 0.5k_{\mathrm{B}}T$ to match the concentration parameter of the von Mises fit in Table S3, and set $\delta = -30°$ to accommodate an equilibrium dihedral angle of 150°.
